# Exploring Facilitators and Barriers to Initiation and Completion of the Human Papillomavirus (HPV) Vaccine Series among Parents of Girls in a Safety Net System

**DOI:** 10.3390/ijerph15020185

**Published:** 2018-01-23

**Authors:** Sean T. O’Leary, Steven Lockhart, Juliana Barnard, Anna Furniss, Miriam Dickinson, Amanda F. Dempsey, Shannon Stokley, Steven Federico, Michael Bronsert, Allison Kempe

**Affiliations:** 1 Department of Pediatrics, University of Colorado School of Medicine, Aurora, CO 80045, USA; Juliana.Barnard@ucdenver.edu (J.B.); AMANDA.DEMPSEY@UCDENVER.EDU (A.F.D.); Allison.Kempe@childrenscolorado.org (A.K.); 2 The Adult and Child Consortium for Health Outcomes Research and Delivery Science, University of Colorado School of Medicine and Children’s Hospital Colorado, Aurora, CO 80045, USA; steven.lockhart@ucdenver.edu (S.L.); alfurniss@yahoo.com (A.F.); miriam.dickinson@ucdenver.edu (M.D.); michael.bronsert@ucdenver.edu (M.B.); 3 Department of Family Medicine, University of Colorado School of Medicine, Aurora, CO 80045, USA; 4 National Center for Immunization and Respiratory Diseases, Centers for Disease Control and Prevention, Atlanta, GA 30329, USA; zma2@cdc.gov; 5 Division of Pediatrics, Denver Health and Hospital Authority, Denver, CO 80204, USA; Steven.Federico@dhha.org; 6 The Colorado School of Public Health, University of Colorado Anschutz Medical Campus, Aurora, CO 80045, USA

**Keywords:** vaccination, human papillomavirus, disparities

## Abstract

*Objective:* To assess, among parents of predominantly minority, low-income adolescent girls who had either not initiated (NI) or not completed (NC) the HPV vaccine series, attitudes and other factors important in promoting the series, and whether attitudes differed by language preference. *Design/Methods:* From August 2013–October 2013, we conducted a mail survey among parents of girls aged 12–15 years randomly selected from administrative data in a Denver safety net system; 400 parents from each group (NI and NC) were targeted. Surveys were in English or Spanish. *Results:* The response rate was 37% (244/660; 140 moved or gone elsewhere; 66% English-speaking, 34% Spanish-speaking). Safety attitudes of NIs and NCs differed, with 40% NIs vs. 14% NCs reporting they thought HPV vaccine was unsafe (*p* < 0.0001) and 43% NIs vs. 21% NCs that it may cause long-term health problems (*p* < 0.001). Among NCs, 42% reported they did not know their daughter needed more shots (English-speaking, 20%, Spanish-speaking 52%) and 39% reported that “I wasn’t worried about the safety of the HPV vaccine before, but now I am” (English-speaking, 23%, Spanish-speaking, 50%). Items rated as very important among NIs in the decision regarding vaccination included: more information about safety (74%), more information saying it prevents cancer (70%), and if they knew HPV was spread mainly by sexual contact (61%). *Conclusions*: Safety concerns, being unaware of the need for multiple doses, and low perceived risk of infection remain significant barriers to HPV vaccination for at-risk adolescents. Some parents’ safety concerns do not appear until initial vaccination.

## 1. Introduction

Attitudinal and logistical factors were identified early on as barriers to human papillomavirus (HPV) vaccination and, despite more than a decade of vaccine availability and recommendation, these barriers persist [1]. In studies done prior to and shortly after introduction of the vaccine in 2006, parents consistently were concerned about vaccine safety, and many expressed a low perceived risk of infection. In addition, in contrast to the other “new” adolescent vaccines at the time, tetanus-diphtheria-acellular pertussis vaccine (Tdap) and meningococcal conjugate vaccine (MCV), the need for three doses for HPV vaccine posed significant logistical challenges, as completion of the series required additional adolescent visits [2]. However, despite the promise of HPV vaccine to prevent cancer, rates of initiation and completion of the series remain low among adolescent girls nationally [3]. 

While barriers to HPV vaccine have been explored, there remain unanswered questions. There have been relatively few studies among parents who had actually declined or delayed vaccination. Also, no prior studies have specifically examined differences in the perspectives of those parents whose daughters had not initiated the series versus those who did not complete it. Moreover, there may be important differences in attitudes about the vaccine by ethnicity and language [4,5] that have not been fully explored. Also, black and Hispanic girls may be less likely to complete the HPV vaccine series than non-Hispanic white girls once they have received a dose [6,7], yet there is little understanding of reasons underlying this phenomenon. We undertook this study to address these gaps in the literature, and to explore and confirm findings from an earlier qualitative study by our group [8]. In that study, we found differences in the attitudes and beliefs of Spanish-speaking and English-speaking parents regarding HPV vaccine, with Spanish-speaking mothers more concerned that accepting the vaccine would encourage sex and English-speaking parents more concerned about vaccine safety. Our objectives were to assess, among parents of predominantly minority, low-income adolescent girls who had either not initiated or not completed the HPV vaccine series, attitudes and other factors important in promoting initiation versus completion of the series, and whether attitudes differed by language preference.

## 2. Methods

### 2.1. Study Design

We conducted a cross-sectional survey among parents of adolescent girls in a large safety net system in Denver, Colorado (Denver Health (DH)). The Colorado Multiple Institutional Review Board (COMIRB) approved this study. 

### 2.2. Study Setting and Population

From August to October 2013, a mail survey was conducted among parents of girls aged 12–15 years randomly selected from administrative data in the DH system. DH serves a population of >17,000 adolescents annually that is predominantly Hispanic, and approximately 40% of the total patient population identify Spanish as their primary language. In 2013, at the time of the study, HPV coverage of ≥1 dose in 13–17 years old girls was 89.8% and coverage for ≥3 doses was 66.8%. The population of girls age 12–15 at the time of the study was 6848. From this population, random samples of 400 parents of girls who were identified as not having received any HPV vaccine doses (non-initiators) and 400 parents of girls who had started but not completed the HPV vaccine series (non-completers) were targeted based on information from the electronic health record. Non-initiators were adolescents with a well-child visit within the previous 2 years but no record of an HPV vaccine in DH administrative data or the state immunization registry. Non-completers were adolescents who had received 1–2 HPV vaccines within the previous 2 years with no record of a 2nd or 3rd dose within 6 or 12 months of the first dose, respectively (or record of subsequent completion of the series). These cutoffs were chosen based on discussions of the study team with CDC (Centers for Disease Control and Prevention) and Denver Health collaborators.

### 2.3. Data Sources

Denver Health has its own immunization registry, VaxTracks, that has been shown to be highly accurate [9]. There are daily uploads to the Colorado Immunization Information System (CIIS), Colorado’s immunization registry. While reporting to CIIS is not mandated in Colorado, >90% of pediatric providers and >70% of family medicine providers report to CIIS. For this study, we first identified the eligible patient population using administrative data from Denver Health. To capture vaccinations received elsewhere, these data were then merged with CIIS data to determine the final study population. The data were extracted from DH administrative records two weeks prior to the launch of the survey and merged with data from CIIS.

### 2.4. Survey Design

The survey was in English or Spanish based on language of preference recorded in the medical record. The language of record is what is used for all DH communications. The survey was based on a combination of qualitative work performed by the study group within the same study population [8] and standardized survey instruments for assessment of parental HPV immunization attitudes and beliefs [10]. All parents were asked demographic questions and 28 questions regarding HPV-related attitudes using 4-point Likert scales (strongly agree to strongly disagree). Parents were also asked to report their daughter’s HPV vaccination status. Parents who responded that their daughter had received 1–2 doses of HPV vaccine were asked a series of nine yes/no questions regarding reasons their daughter had not received a second or third dose followed by a modified four question decisional regret scale on a 4-point Likert scale [11]. All decisional regret questions assumed prior vaccination, such as “Vaccinating my daughter against HPV was the right decision”. Parents who responded that their daughter had never received HPV vaccine were asked to rate the level of importance (very important to very unimportant) of seven factors in helping them decide whether or not to get their daughter vaccinated against HPV. Parents who reported their daughters had already completed the series did not answer questions related to non-initiation or non-completion. The survey was piloted among 6 English- and 6 Spanish-speaking parents and modified based on feedback.

### 2.5. Survey Administration

Parents of non-initiators (n = 400) and non-completers (n = 400) were mailed a pre-letter followed by the survey two weeks later. Non-respondents were sent a reminder postcard and up to two additional surveys over a two-week period. All surveys included cover letters describing that the survey was regarding HPV vaccine and sought to understand their thoughts about HPV vaccine to better understand how parents make decisions above HPV vaccination for their daughters. There were up to two reminder phone calls to non-respondents from a physician within DH recorded in English and Spanish. A $5 bill was included in the initial survey mailing. For surveys that were returned as undeliverable, the study team contacted staff at DH to identify those patients who had moved or gone elsewhere (MOGE) and if confirmed as unreachable, were then removed from the sample. For those undeliverable surveys not identified as MOGE, an Internet search was performed to determine new addresses if available (Intellius, Inc., Bellevue, WA, USA). Initially, 154 surveys were returned as undeliverable or identified by DH staff as MOGE and were searched using Intellius. This search yielded 33 new addresses, and of these, 19 surveys were again returned as undeliverable and 14 surveys were successfully delivered. These 14 were included in the final analysis and the remainder (n = 140) classified as MOGE.

### 2.6. Analytic Methods

Chi-squared test of proportions or Fisher’s Exact test were used for comparisons of characteristics of respondents and non-respondents. When results regarding attitudes were stratified into non-initiators and non-completers, this was based on self-reported vaccination status rather than electronic data, as we hypothesized that parental perception of vaccination status was more relevant to attitudinal responses than actual vaccination status. Results stratified by electronic data are available in an online Appendix A. All statistical analyses were performed using SAS software (SAS 9.3, SAS Institute, Cary, NC, USA).

## 3. Results

### 3.1. Response Rates and Study Sample

The overall response rate was 37% (244/660; 140 MOGE). Respondents were similar to non-respondents in terms of age of the child and language preference (the only variables available for comparison). Overall, 83% of respondents reported being the patient’s mother, 7% the father, 7% the legal guardian, and 3% other. As shown in Table 1, non-initiators were more likely than non-completers to have a college degree, to be White, and to have an income >$50,000. Sixty-six percent of respondents took the survey in English and 34% in Spanish. Respondents to the Spanish-language survey were more likely than respondents to the English-language survey to have public insurance, have less than a high school education, be Hispanic and have income <$20,000/year. 

### 3.2. Differences in Electronic Data and Self-Reported Vaccination Status

**Figure 1:**
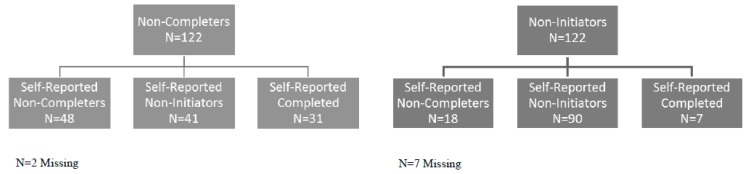
Electronically identified vs. self-reported human papillomavirus vaccination status.

There were significant differences in self-reported vaccination status and vaccination status determined electronically (Figure 1). Based on electronic data, 50% (n = 122) of respondents were non-initiators and 50% (n = 122) were non-completers. Among those who self-reported as non-initiators (n = 131), 69% were non-initiators based on electronic data and 31% were non-completers. Among those who self-reported as non-completers (n = 66), 73% were non-completers based on electronic data and 27% were non-initiators. Six percent of electronically identified non-initiators (n = 7) and 25% of electronically identified non-completers (n = 31) reported they had completed the series. 

### 3.3. Attitudes Regarding HPV Infection and HPV Vaccine

Overall, 62% of parents disagreed that their daughter was at risk for HPV infection. Regarding specific reasons, most parents agreed that their daughter was not at risk for HPV infection because she was not having sexual intercourse (71%). Twenty-four percent of parents agreed that their daughter was only at risk of HPV if she had premarital sex. Few parents (10%) agreed that it was better to get HPV infection than vaccination, or that HPV is not a problem because it can be treated (13%) or because few people have cervical cancer (9%). 

**Figure 2:**
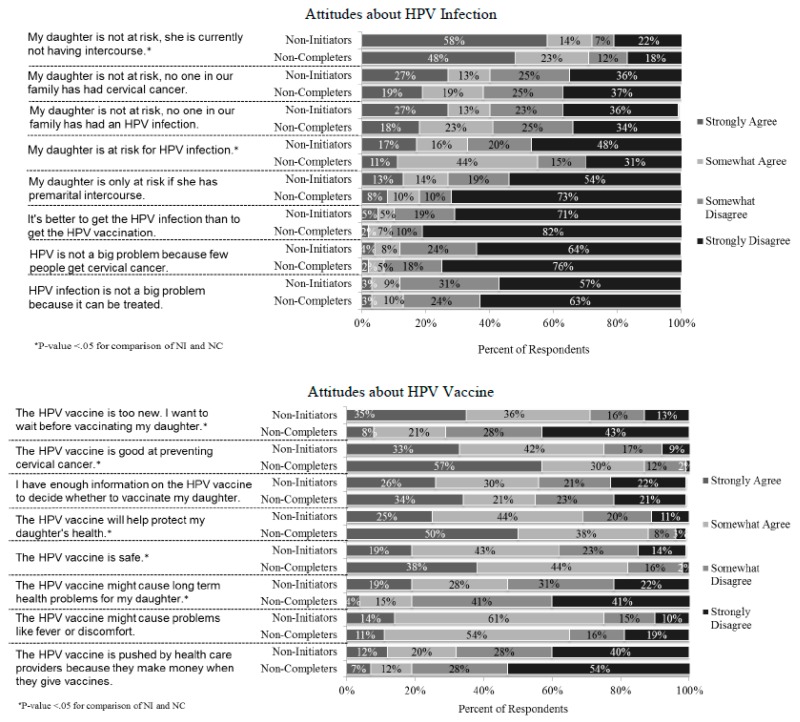
Attitudes about HPV infection and vaccination among parents whose daughters had not started or not completed the Human Papillomavirus (HPV) Vaccine Series. NI: not initiated; NC: not completed.

There were some notable differences in attitudes regarding HPV infection and HPV vaccine between self-reported non-initiators and non-completers (Figure 2). Non-initiators were more likely than non-completers to disagree with the statement “My daughter is at risk for HPV infection” and more likely to agree that “My daughter is not at risk, she is currently not having intercourse”. Parents of non-initiators also had more safety concerns and were less likely to agree that the vaccine is good for protecting their daughter’s health or at preventing cervical cancer. Non-initiators were also more likely to endorse that the HPV vaccine is too new and they wanted to wait before vaccinating their daughter. 

### 3.4. Reasons for Non-Completion and Decisional Regret

Thirty-eight parents reported their daughters had completed the series and were skipped out of questions regarding reasons for non-completion and decisional regret. Among those parents who reported that their daughters had initiated but not completed the HPV vaccine series (n = 40 had started but not completed, n = 11 had started but uncertain of completion), the most commonly reported reasons for not having completed the series were reporting not being due for the next dose yet (52%), not knowing more doses were needed (42%), and reporting that they had not initially had safety concerns about HPV vaccine, but do now (39%). Few parents regretted the decision to vaccinate their daughter (21%) or thought that the HPV vaccine caused their daughter harm (8%). Among these parents who reported their daughter had started but not finished the series (“don’t know/not sure” did not answer this question), 39 out of 40 (98%) reported they planned to complete the series.

### 3.5. Influences on the Decision to Vaccinate

Parents who reported that their daughter had not started the HPV series were asked to rate the importance of several possible influences on the decision of whether or not to vaccinate their daughter against HPV (Table 2). The influences that the highest percentages of parents reported as very important included “If I knew that HPV vaccine can keep my daughter from getting cancer when she is older” (75%), “If there was more information about vaccine safety available” (74%), and “If there were more information saying that the vaccine works really well at preventing cancer” (70%). Of note, of the seven factors queried, “If my daughter’s health care provider strongly recommended it” was the factor least often rated as very important (47%).

### 3.6. Differences in English- and Spanish-Speaking Parents

Items where there were statistically significant differences between English- (ES) and Spanish-speaking (SS) parents of non-initiators are shown in Table 3. Among non-initiators, there were a number of differences in attitudes regarding risk of HPV infection, most notably agreement that their daughter was at risk for HPV infection (ES, 24%, SS, 53%). Regarding attitudes about HPV vaccine, ES non-initiators were more likely than SS to agree that HPV is pushed by health care providers to make money (ES, 41%, SS, 11%) and that it might cause short term side effects (ES, 83%, SS, 56%) and less likely to agree that it is good for protecting their daughter’s health (ES, 63%, SS, 82%). There were fewer differences between ES and SS non-completers, with SS more likely to agree that their daughter is at risk for HPV infection (ES, 46%, SS, 67%, *p* = 0.02). Among non-completers who answered that their daughter had received 1 or 2 doses of HPV vaccine, more SS parents reported that they did not think it was important to get all 3 HPV shots (ES, 20%, SS 52%, *p* = 0.01) and that “I wasn’t worried about the safety of the HPV vaccine before, but now I am” (ES, 23%, SS, 50%, *p* = 0.04).

## 4. Discussion

We report the results of a survey regarding attitudes related to HPV vaccine conducted in an underserved population at high risk for HPV-related disease. We show that safety concerns continue to be a barrier to HPV vaccination, and that there are differences in attitudes of parents who report their daughters have started the series compared to those who have not. We also present new information showing that parents whose daughters have started but not finished the series may acquire safety concerns after initiating the series. Finally, our data show important differences in the reasons for non-initiation and non-completion between Spanish- and English-speaking parents.

Although concerns from parents about the safety of HPV vaccine are not new, two findings from our analysis are cause for concern. First, in 2013, at the time of this survey and a full seven years after licensure of HPV4, there was a great deal more safety data available than there was in the first few years [12,13,14], yet many parents still raised concerns about safety despite extensive efforts by CDC, AAP (American Academy of Pediatrics) and others to publicize the vaccine’s safety record [15,16]. This implies that despite concerted efforts to inform the public about HPV vaccine safety, these messages are not reaching or are not effectively reassuring many parents. Secondly, many parents whose daughters had started the series reported that they had safety concerns only after starting the series, although it is reassuring that most still planned to complete the series. These parents theoretically had had an opportunity to be educated about the safety of the vaccine at the time of the initial vaccination. It is unknown how those concerns developed, but this finding deserves further exploration. In our prior qualitative study, some English- but not Spanish-speaking parents reported that their concerns about HPV vaccine safety developed after their daughters had received the first dose when they began to read or hear things that encourage doubt [8]. With this survey, we confirm that safety concerns developing after initiation is not uncommon, and that it is not limited to English-speakers. This finding is also counter to the supposition that reasons for non-completion of the series are only logistical, whereas reasons for non-initiation are more focused on safety. Our data suggest that efforts to get adolescents to complete the series will involve not only solving logistical issues but also the need for ongoing messaging about the vaccine’s safety among initiators. Another recent study found that child’s fear of needles was a significant reason for non-completion [17], and it may be that parents considered this fear of needles a safety concern since we did not ask about this explicitly. 

Responses among parents who reported their daughters had not started the HPV series provide some focus for vaccine advocates, researchers, and health educators in the creation of future educational interventions to improve vaccination uptake. Similar to prior work, parents request more information about the vaccine’s safety. In addition, it appears there should be more emphasis on cancer prevention. We were somewhat surprised that more parents did not endorse a strong provider recommendation as “very important”, since much of the current messaging around increasing uptake of HPV vaccine is focused on the importance of the provider’s recommendation. A strong provider recommendation was not even in the top five of the possible strategies considered “very important” in the decision to receive the HPV vaccine. While provider recommendation has consistently been shown to influence HPV vaccine uptake [1,18,19], our findings are consistent with a prior study among a predominantly publicly insured population in Florida which showed that parent perception of HPV vaccine safety was strongly influential of vaccine receipt [20]. This finding may also be related to our methods: by providing a list of possible reasons, we may have brought up reasons parents may not have otherwise thought about. For example, the National Immunization Survey-Teen (NIS-Teen) from the same year asked about the most important factors in determining when a teen will receive HPV vaccine in an open-ended fashion, and the most commonly reported reason was lack of provider recommendation [21]. 

An actionable finding from our study, and consistent with our recently published qualitative study [8], is the need to reinforce the message that multiple doses are needed to complete the HPV series among Spanish-speaking parents. In the NIS-Teen, which did not categorize the study population by race/ethnicity, 15% of parents of partially vaccinated girls reported “clinician did not recommend” or “did not know additional shots needed” as a reason for non-completion [21], which is similar to our finding of 20% among English-speakers (versus over half of Spanish speakers). It is also worth noting, though, that the vast majority of parents in the NIS-Teen were not aware that three doses were required at the time of this study. In our study, the reason why knowledge of the need for additional doses differed between Spanish and English-speaking parents is unclear. Most providers within Denver Health, where the study was conducted, speak Spanish, and patient handouts, including educational handouts and Vaccine Information Statements, are available in both English and Spanish. This finding could indicate providers are communicating the need for subsequent doses in English to the English-speaking adolescent, assuming either that the Spanish-speaking parent understands or that the adolescent will tell them if they do not. It is also possible that even though many of the providers at Denver Health speak Spanish, most are not native speakers and may not be comfortable discussing HPV vaccine with the nuance they feel is required. A recent qualitative study offers evidence that this problem is not unique to Denver Health. Investigators in New York found that the primary barrier among Spanish-speaking immigrants was lack of any provider recommendation for the vaccine [22]. Another recent qualitative study from Utah showed that there was confusion about the need for three doses among Spanish-speaking parents [23]. In any case, further work should explore if our finding regarding lack of knowledge regarding completion of the series is common in other settings. Providers should make clear to all parents, English- and Spanish-speaking, that three doses are required for completion of the series. Similarly, with a 2-dose schedule now recommended for those who begin the HPV vaccination series before age 15 [24], the importance of completing two doses will need to be emphasized. It should also be noted that with this study, we were able to examine differences between English- and Spanish-speaking parents, but did not have the power to examine differences by race/ethnicity. Future work should examine attitudes regarding HPV vaccine in specific populations, as there may other important unexplored differences.

Electronic data and self-report are often used to assess vaccination status in research studies. The differences we found between electronic data and self-report have several potential interpretations and implications. For those we identified electronically as non-initiators or non-completers but whose parents reported initiation or completion, respectively, there are at least three possibilities: that their daughters had received the vaccine after the data were pulled but before they answered the survey; that their daughters had received the HPV vaccines but these were not captured electronically; or that they were mistaken and their daughters had not received the vaccines. This last possibility seems less likely as prior work has shown that over-reporting of HPV vaccination is unusual [25]. For those parents who identified their daughters as non-initiators but we identified as having received one or two doses—about 1/3 of the self-reported non-initiators—the interpretation is perhaps more straightforward: it is likely these parents simply did not know their daughter had received the vaccine. It is also possible, however, that some of these parents perceived stigma from admitting to their daughters’ receipt of a vaccine for a sexually-transmitted infection, even if in an anonymous survey. Our findings were similar to the aforementioned study comparing self-reported vaccination status to medical record review, where among 66 mothers whose daughters had received the HPV vaccine according to medical records, 16 reported that they had not [25]. While one could speculate on why this might be, the implication from these data is that researchers should be careful drawing conclusions based only on parental report of vaccination status. 

This study had several strengths and limitations. It is complementary to our recently published qualitative study within the same population [8], confirming several findings that are actionable. This study was also among populations at high risk of not completing the series and of cervical cancer. However, the study focused on one safety net system, and therefore may not be generalizable. Also, survey respondents may have differed from non-respondents. Further, despite extensive efforts, our response rate was sub-optimal, although similar to other surveys in low SES populations. These results must be considered in that context, and further work may be necessary to confirm these findings. This survey was also administered in 2013, so some of these findings may have changed in the interim. In addition, although we asked about educational attainment, we did not measure the literacy rate in the study population, so it is possible that some respondents did not fully understand the questions. It is also unknown if the person answering the survey was the most knowledgeable about the adolescent’s vaccination status, which may explain some of the differences we found between electronic data and self-report. Finally, we performed this study only among girls, and parents of boys may have different attitudes and practices.

## 5. Conclusions

Ongoing safety concerns and low perceived risk of HPV infection remain significant barriers to HPV vaccination among at risk adolescents. Spanish-speaking parents appear to have different barriers than English-speaking parents for initiation of the HPV vaccine, and a concerning proportion of Spanish-speaking parents whose daughters start the series are unaware of the need for subsequent doses. Many parents develop safety concerns about HPV vaccine after starting the series. Providers and health educators should emphasize cancer prevention and vaccine safety when counseling parents and developing educational materials.

## Figures and Tables

**Table 1:** Demographics of HPV survey population *.

Characteristic (%)	All (n = 244)	Non-Completers (n = 122) %	Non-Initiators (n = 122) %	*p*-Value	Survey in English (n = 160, 66%)	Survey in Spanish (n = 84, 34%)	*p*-Value
Child’s age (years):							
12 to <13	30.3	29.5	31.1	0.10	26.2	38.0	0.16
13 to <14	31.5	26.2	36.8		33.1	28.5	
14 to <16	38.1	44.2	31.9		40.6	33.3	
Insurance:							
Private	26.1	20.1	31.4	0.10	37.1	3.7	<0.0001
Public	66.3	73.1	60.3		56.6	86.4	
None	7.4	6.7	8.2		6.2	9.8	
Parent’s Education:							
Less than High School	25.0	31.0	19.1	0.04	13.3	47.5	<0.0001
High School	20.8	21.0	20.8		20.3	21.9	
Some college	27.0	28.5	25.8		28.6	24.3	
College/graduate school	27.0	19.3	34.1		37.5	6.0	
Ethnicity:							
White	20.8	11.6	29.4	0.003	30.1	2.4	<0.0001
Latino/Hispanic	57.5	64.1	51.2		36.5	97.5	
All other	21.6	24.1	19.3		33.3	0.0	
Household Income:							
<$20,000	28.6	36.7	21.0	0.005	24.0	39.3	0.001
$20,000 to <$50,000	46.7	47.9	46.0		44.5	52.4	
≥$50,000	24.6	15.3	33.0		31.3	8.1	

* Table shows vaccination status based on administrative data, not self-report. HPV: human papillomavirus.

**Table 2:** Importance of factors on decision to vaccinate with HPV Vaccine among parents who reported their daughters had never received HPV Vaccine.

Statement	Response	Self-Reported Non-Initiator (n = 131) % (N)
If my daughter’s health care provider strongly recommended it.	Very Important	46.5 (59)
	Somewhat Important	36.2 (46)
	Unimportant	17.3 (22)
If my daughter became sexually active before marriage.	Very Important	58.7 (74)
	Somewhat Important	28.6 (36)
	Unimportant	12.7 (16)
If my daughter’s health care provider said that HPV is spread when kids mess around sexually.	Very Important	48.0 (59)
	Somewhat Important	31.7 (39)
	Unimportant	20.3 (25)
If I knew that HPV is spread mainly through sexual contact.	Very Important	60.8 (76)
	Somewhat Important	27.2 (34)
	Unimportant	12.0 (15)
If I knew that HPV can keep my daughter from getting cancer when she is older.	Very Important	75.0 (93)
	Somewhat Important	16.9 (21)
	Unimportant	8.1 (10)
If there was more information about vaccine safety available.	Very Important	74.4 (93)
	Somewhat Important	18.4 (23)
	Unimportant	7.2 (9)
If there was more information saying that the vaccine works really well at preventing cancer.	Very Important	70.4 (88)
	Somewhat Important	24.8 (31)
	Unimportant	4.8 (6)

**Table 3:** Attitudinal differences between English-speaking and Spanish-Speaking who reported their daughters had not initiated the HPV Vaccine Series.

Statement	Response	English-Speaking (n = 89), % (N)	Spanish-Speaking (n = 38), % (N)	*p*-Value
My daughter is at risk for HPV infection.	Strongly agree	6.8 (6)	39.5 (15)	<0.0001
	Somewhat agree	17.0 (15)	13.2 (5)	
	Somewhat disagree	26.1 (23)	5.3 (2)	
	Strongly disagree	50.0 (44)	42.1 (16)	
My daughter is not at risk for HPV infection because no one in our family has had a HPV infection.	Strongly agree	31.0 (27)	18.9 (7)	0.0225
	Somewhat agree	14.9 (13)	8.1 (3)	
	Somewhat disagree	26.4 (23)	16.2 (6)	
	Strongly disagree	27.6 (24)	56.8 (21)	
My daughter is not at risk for HPV infection because no one in our family has cervical cancer.	Strongly agree	30.7 (27)	18.4 (7)	0.0276
	Somewhat agree	14.8 (13)	7.9 (3)	
	Somewhat disagree	27.3 (24)	18.4 (7)	
	Strongly disagree	27.3 (24)	55.3 (21)	
My daughter is not at risk for HPV infection because she is not currently having sexual intercourse.	Strongly agree	68.6 (59)	34.2 (13)	0.0001
	Somewhat agree	15.1 (13)	10.5 (4)	
	Somewhat disagree	4.7 (4)	10.5 (4)	
	Strongly disagree	11.6 (10)	44.7 (17)	
HPV infection is not a big problem because not very many people get cervical cancer.	Strongly agree	1.1 (1)	10.8 (4)	0.0164
	Somewhat agree	8.0 (7)	8.1 (3)	
	Somewhat disagree	29.9 (26)	10.8 (4)	
	Strongly disagree	60.9 (53)	70.3 (26)	
The HPV vaccine is pushed by health care providers because they make money when they give vaccines.	Strongly agree	14.8 (13)	5.4 (2)	0.0091
	Somewhat agree	26.1 (23)	5.4 (2)	
	Somewhat disagree	26.1 (23)	32.4 (12)	
	Strongly disagree	33.0 (29)	56.8 (21)	
The HPV vaccine is good at preventing cervical cancer.	Strongly agree	23.0 (20)	55.3 (21)	0.0057
	Somewhat agree	47.1 (41)	28.9 (11)	
	Somewhat disagree	19.5 (17)	10.5 (4)	
	Strongly disagree	10.3 (9)	5.3 (2)	
The HPV vaccine might cause short-term problems like fever.	Strongly agree	17.2 (15)	5.6 (2)	0.0096
	Somewhat agree	65.5 (57)	50.0 (18)	
	Somewhat disagree	11.5 (10)	25.0 (9)	
	Strongly disagree	5.7 (5)	19.4 (7)	
The HPV vaccine is good for protecting my daughter’s health.	Strongly agree	17.2 (15)	42.1 (16)	0.0125
	Somewhat agree	46.0 (40)	39.5 (15)	
	Somewhat disagree	25.3 (22)	7.9 (3)	
	Strongly disagree	11.5 (10)	10.5 (4)	
I trust what my health care provider tells me about vaccines.	Strongly agree	33.7 (30)	60.5 (23)	0.0433
	Somewhat agree	47.2 (42)	26.3 (10)	
	Somewhat disagree	13.5 (12)	7.9 (3)	
	Strongly disagree	5.6 (5)	5.3 (2)	

## Abbreviations

CI	Confidence Interval
AAP	American Academy of Pediatrics
CDC	Centers for Disease Control and Prevention

## Appendix A

**Table A1:** Attitudinal differences between electronically identified non-completers and non-initiators.

Statement	Response	Non Completers (n = 122), % (N)	Non Initiators (n = 122), % (N)	*p*-Value
My daughter is at risk for HPV infection.	Strongly agree	15.8 (19)	15.8 (19)	0.3970
	Somewhat agree	28.3 (34)	19.2 (23)	
	Somewhat Disagree	17.5 (21)	20.8 (25)	
	Strongly Disagree	38.3 (46)	44.2 (53)	
My daughter is not at risk for HPV infection because no one in our family has had a HPV infection.	Strongly agree	25.2 (30)	25.2 (30)	0.6677
	Somewhat agree	17.6 (21)	13.4 (16)	
	Somewhat Disagree	22.7 (27)	20.2 (24)	
	Strongly Disagree	34.5 (41)	41.2 (49)	
My daughter is not at risk for HPV infection because no one in our family has cervical cancer.	Strongly agree	26.4 (32)	24.8 (30)	0.9792
	Somewhat agree	14.9 (18)	14.0 (17)	
	Somewhat Disagree	21.5 (26)	21.5 (26)	
	Strongly Disagree	37.2 (45)	39.7 (48)	
My daughter is not at risk for HPV infection because she is not currently having sexual intercourse.	Strongly agree	50.4 (60)	60.5 (72)	0.3548
	Somewhat agree	16.0 (19)	15.1 (18)	
	Somewhat Disagree	9.2 (11)	8.4 (10)	
	Strongly Disagree	24.4 (29)	16.0 (19)	
It is better to get the HPV infection than to get the HPV vaccination.	Strongly agree	4.2 (5)	4.2 (5)	0.9299
	Somewhat agree	5.1 (6)	6.7 (8)	
	Somewhat Disagree	13.6 (16)	15.1 (18)	
	Strongly Disagree	77.1 (91)	73.9 (88)	
My daughter is only at risk for HPV infection if she has premarital intercourse.	Strongly agree	11.6 (14)	9.2 (11)	0.7845
	Somewhat agree	14.0 (17)	12.5 (15)	
	Somewhat Disagree	15.7 (19)	20.0 (24)	
	Strongly Disagree	58.7 (71)	58.3 (70)	
HPV infection is not a big problem because not very many people get cervical cancer.	Strongly agree	2.5 (3)	3.3 (4)	0.8862
	Somewhat agree	7.5 (9)	5.8 (7)	
	Somewhat Disagree	20.8 (25)	23.3 (28)	
	Strongly Disagree	69.2 (83)	67.5 (81)	
HPV infection is not a big problem because it can be treated.	Strongly agree	6.6 (8)	3.4 (4)	0.7237
	Somewhat agree	9.1 (11)	7.8 (9)	
	Somewhat Disagree	25.6 (31)	27.6 (32)	
	Strongly Disagree	58.7 (71)	61.2 (71)	
I have enough information on the HPV vaccine to decide whether to vaccinate my daughter.	Strongly agree	32.8 (39)	29.5 (36)	0.3699
	Somewhat agree	26.1 (31)	27.9 (34)	
	Somewhat Disagree	23.5 (28)	17.2 (21)	
	Strongly Disagree	17.6 (21)	25.4 (31)	
The HPV vaccine is pushed by health care providers because they make money when they give vaccines.	Strongly agree	9.3 (11)	10.7 (13)	0.3169
	Somewhat agree	13.6 (16)	17.4 (21)	
	Somewhat Disagree	25.4 (30)	32.2 (39)	
	Strongly Disagree	51.7 (61)	39.7 (48)	
The HPV vaccine is good at preventing cervical cancer.	Strongly agree	46.7 (56)	38.7 (46)	0.3670
	Somewhat agree	38.3 (46)	37.8 (45)	
	Somewhat Disagree	10.8 (13)	16.8 (20)	
	Strongly Disagree	4.2 (5)	6.7 (8)	
The HPV vaccine might cause problems like fever or discomfort.	Strongly agree	11.2 (13)	13.7 (16)	0.1991
	Somewhat agree	50.0 (58)	60.7 (71)	
	Somewhat Disagree	24.1 (28)	15.4 (18)	
	Strongly Disagree	14.7 (17)	10.3 (12)	
The HPV vaccine might cause long-term health problems for my daughter.	Strongly agree	7.1 (8)	16.2 (19)	0.0007
	Somewhat agree	14.2 (16)	27.4 (32)	
	Somewhat Disagree	36.3 (41)	35.0 (41)	
	Strongly Disagree	42.5 (48)	21.4 (25)	
The HPV vaccine is safe.	Strongly agree	44.1 (52)	16.5 (19)	<0.0001
	Somewhat agree	41.5 (49)	43.5 (50)	
	Somewhat Disagree	11.0 (13)	25.2 (29)	
	Strongly Disagree	3.4 (4)	14.8 (17)	
The HPV vaccine is too new. I want to wait before vaccinating my daughter.	Strongly agree	10.0 (12)	36.7 (44)	<0.0001
	Somewhat agree	29.2 (35)	29.2 (35)	
	Somewhat Disagree	23.3 (28)	20.0 (24)	
	Strongly Disagree	37.5 (45)	14.2 (17)	
The HPV vaccine will help protect my daughter’s health.	Strongly agree	47.1 (57)	27.1 (32)	0.0059
	Somewhat agree	38.0 (46)	44.1 (52)	
	Somewhat Disagree	9.1 (11)	19.5 (23)	
	Strongly Disagree	5.8 (7)	9.3 (11)	
I trust what my health care provider tells me about vaccines.	Strongly agree	66.9 (81)	38.0 (46)	0.0001
	Somewhat agree	26.4 (32)	43.0 (52)	
	Somewhat Disagree	3.3 (4)	13.2 (16)	
	Strongly Disagree	3.3 (4)	5.8 (7)	
Has your daughter ever received the HPV vaccine?	No	34.7 (41)	78.3 (90)	<0.0001
	Yes	65.3 (77)	21.7 (25)	
For each reason below, please answer YES if this is a reason that your daughter has not gotten all 3 shots. Please answer NO if this is not a reason that your daughter has not gotten all 3 shots. (Please check YES or NO for each reason)				
I didn’t know she needed another HPV shot.	Yes	38.8 (19)	52.9 (9)	0.3086
	No	61.2 (30)	47.1 (8)	
I didn’t think it was important for her to get all 3 HPV shots.	Yes	36.0 (18)	46.7 (7)	0.4564
	No	64.0 (32)	53.3 (8)	
I wasn’t worried about the safety of the HPV vaccine until now.	Yes	34.7 (17)	53.3 (8)	0.1954
	No	65.3 (32)	46.7 (7)	
She is not yet due for the next HPV shot.	Yes	51.0 (25)	57.1 (8)	0.6858
	No	49.0 (24)	42.9 (6)	
Please tell us how much you agree or disagree with each of the following statements about your decision to vaccinate your daughter against HPV.				
Vaccinating my daughter against HPV was the right decision.	Strongly agree	70.9 (39)	42.1 (8)	0.0410
	Somewhat agree	23.6 (13)	36.8 (7)	
	Somewhat Disagree	5.5 (3)	21.1 (4)	
	Strongly Disagree	0	0	
I regret vaccinating my daughter against HPV.	Strongly agree	3.8 (2)	10.5 (2)	0.2415
	Somewhat agree	13.2 (7)	21.1 (4)	
	Somewhat Disagree	17.0 (9)	26.3 (5)	
	Strongly Disagree	66.0 (35)	42.1 (8)	
Vaccinating my daughter against HPV caused her harm.	Strongly agree	3.7 (2)	0.0 (0)	0.2493
	Somewhat agree	5.6 (3)	5.3 (1)	
	Somewhat Disagree	16.7 (9)	36.8 (7)	
	Strongly Disagree	74.1 (40)	57.9 (11)	
I would choose not to vaccinate my daughter against HPV if I had to do it over again.	Strongly agree	3.7 (2)	5.0 (1)	0.4359
	Somewhat agree	7.4 (4)	10.0 (2)	
	Somewhat Disagree	16.7 (9)	30.0 (6)	
	Strongly Disagree	72.2 (39)	55.0 (11)	
Tell us how important each of the following would be in helping you to decide if your daughter should get the HPV vaccine or not.				
If my daughter’s health care provider strongly recommended it.	Very Important	69.1 (56)	42.1 (48)	0.0005
	Somewhat Important	25.9 (21)	36.8 (42)	
	Somewhat Unimportant	3.7 (3)	8.8 (10)	
	Very Unimportant	1.2 (1)	12.3 (14)	
If my daughter became sexually active before marriage.	Very Important	65.0 (52)	56.6 (64)	0.4598
	Somewhat Important	25.0 (20)	26.5 (30)	
	Somewhat Unimportant	6.3 (5)	8.0 (9)	
	Very Unimportant	3.8 (3)	8.8 (10)	
If my daughter’s health care provider said that HPV is spread when kids mess around sexually.	Very Important	63.0 (51)	41.8 (46)	0.0190
	Somewhat Important	25.9 (21)	33.6 (37)	
	Somewhat Unimportant	7.4 (6)	12.7 (14)	
	Very Unimportant	3.7 (3)	11.8 (13)	
If I knew that HPV is spread mainly through sexual contact.	Very Important	69.5 (57)	55.0 (61)	0.0051
	Somewhat Important	29.3 (24)	28.8 (32)	
	Somewhat Unimportant	0.0 (0)	10.8 (12)	
	Very Unimportant	1.2 (1)	5.4 (6)	
If I knew that HPV can keep my daughter from getting cancer when she is older.	Very Important	76.5 (62)	76.6 (85)	0.0075
	Somewhat Important	23.5 (19)	12.6 (14)	
	Somewhat Unimportant	0.0 (0)	6.3 (7)	
	Very Unimportant	0.0 (0)	4.5 (5)	
If there was more information about vaccine safety available.	Very Important	75.6 (62)	73.0 (81)	0.1205
	Somewhat Important	23.2 (19)	18.0 (20)	
	Somewhat Unimportant	1.2 (1)	6.3 (7)	
	Very Unimportant	0.0 (0)	2.7 (3)	
If there was more information saying that the vaccine works really well at preventing cancer.	Very Important	79.5 (66)	73.0 (81)	0.0996
	Somewhat Important	20.5 (17)	19.8 (22)	
	Somewhat Unimportant	0.0 (0)	4.5 (5)	
	Very Unimportant	0.0 (0)	2.7 (3)	

**Table A2:** Comparison of respondents based on self-report, including non-initiation, non-completion, and completion of the human papillomavirus vaccine series.

Question	SR Non-Initiator	SR Non-Completer	SR Completer	*p*-Value (SRC vs. SRNI)	*p*-Value (SRC vs. SRNC)	*p*-Value (SRNI vs. SRNC)
	n = 131	n = 66	n = 38			
	% (N)	% (N)	% (N)			
My daughter is at risk for HPV infection.				0.64	0.12	<0.01
Strongly agree	16.0% (21)	10.9% (7)	22.9% (8)			
Somewhat agree	15.3% (20)	42.2% (27)	20.0% (7)			
Somewhat Disagree	22.1% (29)	15.6% (10)	17.1% (6)			
Strongly Disagree	46.6% (61)	31.3% (20)	40.0% (14)			
My daughter is not at risk, no one in our family has had an HPV infection.				0.59	0.42	0.19
Strongly agree	27.9% (36)	17.5% (11)	22.2% (8)			
Somewhat agree	13.2% (17)	23.8% (15)	13.9% (5)			
Somewhat Disagree	23.3% (30)	23.8% (15)	16.7% (6)			
Strongly Disagree	35.7% (46)	34.9% (22)	47.2% (17)			
My daughter is not at risk, no one in our family has had cervical cancer.				0.1	0.13	0.4
Strongly agree	27.5% (36)	18.5% (12)	25.0% (9)			
Somewhat agree	13.0% (17)	20.0% (13)	13.9% (5)			
Somewhat Disagree	25.2% (33)	24.6% (16)	8.3% (3)			
Strongly Disagree	34.4% (45)	36.9% (24)	52.8% (19)			
My daughter is not at risk, she is currently not having intercourse.				0.48	0.74	0.31
Strongly agree	58.1% (75)	49.2% (31)	50.0% (18)			
Somewhat agree	14.7% (19)	22.2% (14)	13.9% (5)			
Somewhat Disagree	6.2% (8)	11.1% (7)	13.9% (5)			
Strongly Disagree	20.9% (27)	17.5% (11)	22.2% (8)			
It is better to get the HPV infection than to get the HPV vaccination.				0.1	0.28	0.13
Strongly agree	4.7% (6)	1.6% (1)	8.1% (3)			
Somewhat agree	4.7% (6)	7.9% (5)	2.7% (1)			
Somewhat Disagree	20.5% (26)	9.5% (6)	5.4% (2)			
Strongly Disagree	70.1% (89)	81.0% (51)	83.8% (31)			
My daughter only at risk if she has premarital intercourse.				0.9	0.46	0.14
Strongly agree	12.3% (16)	7.8% (5)	8.1% (3)			
Somewhat agree	14.6% (19)	10.9% (7)	13.5% (5)			
Somewhat Disagree	20.0% (26)	10.9% (7)	21.6% (8)			
Strongly Disagree	53.1% (69)	70.3% (45)	56.8% (21)			
HPV not a big problem because few people get cervical cancer.				0.08	0.63	0.32
Strongly agree	3.9% (5)	1.6% (1)	2.7% (1)			
Somewhat agree	8.5% (11)	4.7% (3)	2.7% (1)			
Somewhat Disagree	26.4% (34)	18.8% (12)	10.8% (4)			
Strongly Disagree	61.2% (79)	75.0% (48)	83.8% (31)			
HPV infection is not a big problem because it can be treated.				0.17	0.53	0.73
Strongly agree	4.0% (5)	3.1% (2)	8.1% (3)			
Somewhat agree	9.5% (12)	9.4% (6)	5.4% (2)			
Somewhat Disagree	31.0% (39)	23.4% (15)	16.2% (6)			
Strongly Disagree	55.6% (70)	64.1% (41)	70.3% (26)			
I have enough information on the HPV vaccine to decide whether to vaccinate my daughter.				<0.01	0.06	0.48
Strongly agree	25.2% (33)	33.3% (21)	51.4% (19)			
Somewhat agree	29.8% (39)	20.6% (13)	29.7% (11)			
Somewhat Disagree	21.4% (28)	23.8% (15)	10.8% (4)			
Strongly Disagree	23.7% (31)	22.2% (14)	8.1% (3)			
The HPV vaccine is pushed by health care providers because they make money when they give vaccines.				0.31	0.99	0.26
Strongly agree	11.5% (15)	7.9% (5)	8.3% (3)			
Somewhat agree	20.0% (26)	11.1% (7)	8.3% (3)			
Somewhat Disagree	28.5% (37)	28.6% (18)	30.6% (11)			
Strongly Disagree	40.0% (52)	52.4% (33)	52.8% (19)			
The HPV vaccine is good at preventing cervical cancer.				0.02	0.54	<0.01
Strongly agree	32.3% (42)	56.5% (35)	56.8% (21)			
Somewhat agree	43.1% (56)	29.0% (18)	37.8% (14)			
Somewhat Disagree	16.2% (21)	12.9% (8)	5.4% (2)			
Strongly Disagree	8.5% (11)	1.6% (1)				
The HPV vaccine might cause problems like fever or discomfort.				0.23	0.68	0.27
Strongly agree	13.3% (17)	10.2% (6)	16.2% (6)			
Somewhat agree	61.7% (79)	52.5% (31)	43.2% (16)			
Somewhat Disagree	15.6% (20)	18.6% (11)	24.3% (9)			
Strongly Disagree	9.4% (12)	18.6% (11)	16.2% (6)			
The HPV vaccine might cause long-term health problem for my daughter.				<0.01	0.52	<0.01
Strongly agree	18.1% (23)	3.6% (2)	2.7% (1)			
Somewhat agree	28.3% (36)	14.3% (8)	8.1% (3)			
Somewhat Disagree	31.5% (40)	41.1% (23)	32.4% (12)			
Strongly Disagree	22.0% (28)	41.1% (23)	56.8% (21)			
The HPV vaccine is safe.				<0.01	0.06	<0.01
Strongly agree	19.2% (25)	38.6% (22)	59.5% (22)			
Somewhat agree	43.8% (57)	43.9% (25)	37.8% (14)			
Somewhat Disagree	23.1% (30)	15.8% (9)	2.7% (1)			
Strongly Disagree	13.8% (18)	1.8% (1)				
The HPV vaccine is too new. I want to wait before vaccinating daughter.				<0.01	0.76	<0.01
Strongly agree	34.6% (45)	7.9% (5)	5.4% (2)			
Somewhat agree	36.9% (48)	23.8% (15)	16.2% (6)			
Somewhat Disagree	16.2% (21)	27.0% (17)	27.0% (10)			
Strongly Disagree	12.3% (16)	41.3% (26)	51.4% (19)			
The HPV vaccine will help protect my daughter’s health.				<0.01	0.85	<0.01
Strongly agree	24.6% (32)	50.0% (31)	59.5% (22)			
Somewhat agree	45.4% (59)	38.7% (24)	32.4% (12)			
Somewhat Disagree	19.2% (25)	8.1% (5)	5.4% (2)			
Strongly Disagree	10.8% (14)	3.2% (2)	2.7% (1)			
I trust what my health care provider tells me about vaccines.				<0.01	0.81	0.06
Strongly agree	40.9% (54)	60.9% (39)	72.2% (26)			
Somewhat agree	42.4% (56)	31.3% (20)	22.2% (8)			
Somewhat Disagree	11.4% (15)	4.7% (3)	2.8% (1)			
Strongly Disagree	5.3% (7)	3.1% (2)	2.8% (1)			

Note: SRNI, self-reported non-initiator; SRNC, self-reported non-completer; SRC, self-reported completer; HPV, human papillomavirus.
